# John Keats, Apothecary and Poet

**Published:** 1921-10

**Authors:** 


### THE REVIEW OF THE MONTH.

### JOHN KEATS: APOTHECARY AND POET.

One hundred years ago John Keats died in Rome. He was only twenty-five years old, but he came nearer than other mortals to perfection during his short length of days. Some of the great poets have known that their verse would be immortal, and that their work, like Keats' own "Nightingale," was not born for death; but this fore-knowledge, rather than giving solace to the poet, has brought with it the heavy sense of responsibility, lest anything common or unclean should pass among his jewels down the ages. Spenser, to whom Keats owed so much, knew that his epithalamion was "for short time an endless monument"; Shakespeare knew well that his poetry would live; and a later poet, who, like Keats, was instructed in the art of medicine, wrote that his love and the flower that she gave to him would never wither, "sheltered and shut in a nook of rhyme from the reaper man and his reaper Time." Keats also, despite his humility, had this prescience of immortality: "I think," he said, "I shall be among the English poets after my death." He knew that Time's fell hand would touch him only lightly.

He is, above others, the Poet of Beauty—of Beauty in Nature, in the heart of man, and in the written word. Even as physical beauty was so longed for and prized by the Greeks of old time, so he, we may fancy, had he been given the choice, would rather have written a fair poem than have worn the crown of a king. It was no earthly beauty, however, that would content him; but that eternal loveliness of thought and word at which the poet arrives, so hardly, after long travail and pain. His reward is the gratitude of all those who delight in things that are fair and lovely; and he, in the elysium of the souls of poets, will be found among those few who have lightened the burden of the world's unhappiness.

It is fitting in this centenary year that the works of the apothecary-poet should receive an appreciation from the pen of one belonging to his order. Sir George Newman, who is himself an honorary Freeman of the Society of Apothecaries of London, has given lately to the world of letters an essay on Keats,* which is full of the insight and sympathy which alone could be bestowed by one doctor on the work of another. The best essay on Shelley was the work of a brother poet, Francis Thompson; and the most intimate appreciation of the life and work of Keats is that recently written by his brother physician. That apothecary of a hundred years ago and this physician of to-day are members not only of the same profession, but also of that small company who know and love the significant tone of words and delight in the beauty of writing. Sir George Newman, an exponent of medical and literary art, has become the interpreter of the apothecary-poet; and we could have thought of none better able to direct us.

"It is impossible," he says, "to believe that a medical training of six years had left no enduring effect on a sensitive and responsive mind like that of John Keats. Can the study of medicine ever leave a man where it finds him? Whether he practises his profession or not, is he not marked for life? Does he not in any case at least bear the mark in the immediately succeeding years? Do we not find it to be so in Schiller, Goldsmith, and Crabbe . . . ? At the end of his six years of medicine Keats had but four more to live, and some things certainly he had learned which were not without effect on those remaining years. . . . A born lover of Nature, he became a trained and acute observer, and his poems and letters supply abundant proof of it." In his apprenticeship to an apothecary at Edmonton, and later in his student days at Guy's Hospital, he learned "the larger exercise of compassion toward human suffering and love of human kind"; and he came to know, taught perhaps by his own frail body, the parts that health and disease play in human affairs. "Banish health," he says, "and banish all the world."

We shall never know what treasures of poetry he took with him to the grave; but we, his heirs to-day, cannot complain that he left us a meagre heritage. Had he but given us only one ode—nay, even a line or two of his perfection—it would have been enough to stir the souls of men. There are poets who have left behind them, amid much dross and worthlessness, some few lines of winged and magic words—Donne, Webster, Poe, and Coleridge are among that number; but no other poets, except Shakespeare and Keats, have shown us so much fine gold in their work, interwoven with so little that is unworthy and base. We have no need to search diligently in order to discover his riches. It is perhaps in the "Ode to the Nightingale," that Keats comes most nearly to absolute perfection of word and tune:

> Thou wast not born for death, immortal Bird!
> No hungry generations tread thee down;
> The voice I hear this passing night was heard
> In ancient days by emperor and clown;
> Perhaps the self-same song that found a path
> Through the sad heart of Ruth, when sick for home,
> She stood in tears amid the alien corn;
> The same that oft-times hath
> Charm'd magic casements, opening on the foam
> Of perilous seas, in faery lands forlorn.

No other poet could have written this, nor dreamed, even, the last two lines. We cannot wonder, as Sir George Newman says, that "this Ode has been described by a competent critic as 'one of the final masterpieces of human work in all time and for all ages.'" Yet no criticism or praise, however just, can add anything to the perfection of the poet's singing, nor illumine that beauty to which he was ever striving and was so often able to attain—that beauty by the means of

* "John Keats: Apothecary and Poet." By Sir George Newman, K.C.B., M.D., D.C.L. Published by T. Booth, St. Peter's Close, Sheffield, 1s. 36 pages.

**The Review of the Month—(*cont.*).**

which he opened to the imprisoned mind so many doors on the wide spaces of Nature.

His latest essayist writes of him: "In his apprehension of Beauty, Truth, and Love Keats had a foresight of the most enlightened modern social thought, whether scientific or altruistic. A recognition of his doctrine, it has been well said, would have meant a new life in society. It may do that even yet. . . . He became the seer and forth-teller of the Gospel of Beauty, a prophet of Faith and Hope and Love—for the soul and for the nation, a prophet who, in Shelley's noble, memorial words in *Adonais*, 'outsoared the shadow of our night.'"

